# Synthesis and Characterization of Organic-Inorganic Nanocomposite Poly-o-anisidine Sn(IV) Arsenophosphate: Its Analytical Applications as Pb(II) Ion-Selective Membrane Electrode

**DOI:** 10.1155/2009/659215

**Published:** 2009-08-09

**Authors:** Asif Ali Khan, Umme Habiba, Anish Khan

**Affiliations:** Analytical and Polymer Research Laboratory, Department of Applied Chemistry, Faculty of Engineering and Technology, Aligarh Muslim University, Aligarh 202002, India

## Abstract

Poly-o-anisidine Sn(IV) arsenophosphate is a newly synthesized nanocomposite material and has been characterized on the basis of its chemical composition, ion exchange capacity, TGA-DTA, FTIR, X-RAY, SEM, and TEM studies. On the basis of distribution studies, the exchanger was found to be highly selective for lead that is an environmental pollutant. For the detection of lead in water a heterogeneous precipitate based ion-selective membrane electrode was developed by means of this composite cation exchanger as electroactive material. The membrane electrode is mechanically stable, with a quick response time, and can be operated over a wide pH range. The selectivity coefficients were determined by mixed solution method and revealed that the electrode is sensitive for Pb(II) in presence of interfering cations. The practical utility of this membrane electrode has been established by employing it as an indicator electrode in the potentiometric titration of Pb(II).

## 1. Introduction

During the last 15 years, the development of organic/inorganic hybrid materials has been an important enterprise for people from very diverse origins (academic, research and industrial sectors). The intrinsic multifunctional character of these materials makes them potentially useful in multiple fields. Illustrative examples of this versatility are their high-added-value applications as coatings for corrosion protection and abrasion resistance, artificial membranes for ultra- and nanofiltration, pervaporation and gas separation, catalysts and nanoscopic reactors, adsorbents of toxic compounds, biomaterials for osteo-reconstructive surgery or ophthalmic, materials with specific optic, electrical and/or magnetic properties for telecommunications or information displays, and so forth. The growing interest of this subject matter has been reflected in two symposia “organized recently by the Materials Research Society [1, 2]”. Most of the organic/inorganic hybrid materials are nanocomposite materials in which the inorganic part and the organic entities interact at molecular level in the nanoscopic domain. This kind of materials often present the best properties of each of its components in a synergic way, offering a unique opportunity to prepare tailor-made new materials with chemical, physical, and mechanical properties of a high performance as discussed elsewhere [3, 4].

 The most obvious advantage of organic and inorganic hybrids is that they can favorably combine the often dissimilar properties of organic and inorganic components in one material. Hybrid materials represent one of the most fascinating developments in material chemistry in recent years. Thus efforts have been made to synthesize such hybrid ion exchangers with good ion exchange properties, high stability, reproducibility, and selectivity for heavy toxic metal ions as discussed by Khan et al. [5]. In the present research work Poly-o-anisidine Sn(IV) arsenophosphate, is a newly synthesized organic-inorganic nanocomposite material developed in our laboratory that possessed all such characteristics discussed earlier and is highly selective for lead, a hazardous toxic material in the environment. The material is also used in making Pb(II) ion selective membrane electrode.

## 2. Experimental

### 2.1. Chemicals and Reagents

The main reagents used for the synthesis were obtained from CDH, Loba Chemie, and E-Merck (India Ltd., used as received). All other reagents and chemicals were of analytical grade. The following instruments were used for various studies made for chemical analysis and characterization of the composite material: UV/VIS spectrophotometer (Elico, India), model EI 301 E; a thermal analyzer (V2.2A DuPont 9900); Elemental analyzer-Elementary Vario EL III, Carlo-Erba, model 1108; a scanning electron microscope-LEO 435 VP (Australia); FTIR spectrometer (Perkin Elmer, USA), model Spectrum BX; an X-ray diffractometer (Phillips, Holland), model PW 1148/89 with Cu K*α* radiations; an automatic temperature controlled water bath incubator shaker (Elcon, India); a digital potentiometer (Equiptronics EQ 609, India); accuracy ±  0.1 mV with a saturated calomel electrode as reference electrode; an electronic balance (digital) (Sartorius, Japan), model 21 OS, Japan.

### 2.2. Preparation of Reagent Solutions

0.1 molL^−1^  solution of Tin tetrachloride, SnCl_4_ · 5H_2_O, and O-phosphoric acid (H_3_PO_4_) were prepared in 1 molL^−1^HCl and demineralized water (DMW), respectively. 0.1 molL^−1^ solution of disodium arsenate, Na_2_HAsO_4_ · 7H_2_O was prepared in DMW. 2.5% solution of orthoanisidine, CH_3_OC_6_H_4_NH_2_, and 0.05 molL^−1^ solution of ammonium persulphate (NH_4_)_2_S_2_O_8_ were prepared in 1 molL^−1^ HCl.

### 2.3. Preparation of Poly-o-anisidine Sn(IV) Arsenophosphate Nanocomposite

#### 2.3.1. Synthesis of Poly-o-anisidine

Poly-o-anisidine is oxidatively synthesized using ammonium persulphate (NH_4_)_2_S_2_O_8_ under the controlled condition as discussed by Ram et al. [6]. The organic polymer derivative of o-methoxy aniline (poly-o-anisidine) was prepared by mixing in similar volume ratios of the solution of 0.05 molL^−1^ ammonium persulphate prepared in 1 molL^−1^  HCl and 2.5% distilled o-anisidine prepared in 1 molL^−1^ HCl with continuous stirring by a magnetic stirrer for 2 hours at 0°C. A green colored gel was obtained. The gel was kept for 24 hours at room temperature.

#### 2.3.2. Synthesis of Sn(IV) Arsenophosphate

The inorganic precipitate of Sn(IV) arsenophosphate ion-exchanger was prepared at room temperature (25 ± 2°C) by mixing an aqueous solution of 0.1 molL^−1^ o-phosphoric acid (H_3_PO_4_) to aqueous solutions of 0.1 molL^−1^ disodium arsenate (Na_2_HAsO_4_ · 7H_2_O) and 0.1 molL^−1^ Tin tetrachloride in 1 molL^−1^   HCl in different mixing volume ratios as discussed by Niwas et al. [7]. The white precipitates were obtained, when the pH of the mixtures was adjusted to ~1 by adding aqueous ammonia with constant stirring. 

#### 2.3.3. Synthesis of Poly-o-anisidine Sn(IV) Arsenophosphate.

The composite cation-exchanger was prepared by the sol-gel mixing of poly-o-anisidine, an organic polymer, into the inorganic precipitate of Sn(IV) arsenophosphate. In this process, when the gel of poly-o-anisidine were added to the white inorganic precipitate of Sn(IV) arsenophosphate with a constant stirring, the resultant mixture was turned slowly into a black colored slurries. The resultant black colored slurries were kept for 24 hours at room temperature (25 ± 2°C). Now the poly-o-anisidine-based composite cation-exchanger gels were filtered off, washed thoroughly with DMW to remove excess acid and any adhering trace of ammonium persulphate. The washed gels were dried over P_4_O_10_ at 45°C in an oven. The dried products were immersed in DMW to obtain small granules. They were converted to the H^+^ form by keeping it in 1 molL^−1^  HNO_3_ solution for 24 hours with occasional shaking intermittently replacing the supernatant liquid. The excess acid was removed after several washing with DMW. The materials were finally dried at 40°C and sieved to obtain particles of particular size range (~125 *μ*m). Hence a number of poly-o-anisidine Sn(1V) arsenophosphate nanocomposite cation-exchanger samples were prepared, and on the basis of Na^+^ exchange capacity (I.E.C), percent of yield, and physical appearance, sample (S-9) was selected for further studies.

### 2.4. Chemical Composition

The chemical composition of poly-o-anisidine Sn(IV) arsenophosphate composite cation exchanger (S-9) was determined by using elemental analyzer, inductively coupled plasma mass spectrophotometer and UV-visible spectrophotometer for CHN, As, Sn, and P.

### 2.5. Scanning Electron Microscopy (SEM) Studies

 Microphotographs of the original form of poly-o-anisidine (S-5), inorganic precipitate of Sn(IV) arsenophosphate (S-1), and organic-inorganic composite material poly-o-anisidine Sn(IV) arsenophosphate (S-9) were obtained by the scanning electron microscope at various magnifications.

### 2.6. X-Ray Analysis

 Powder X-ray diffraction (XRD) pattern was obtained in an aluminum sample holder for poly-o-anisidine Sn(IV) arsenophosphate (S-9) in the original form using a PW, 1148/89-based diffractometer with Cu K*α* radiations.

### 2.7. Fourier Transform Infra Red (FTIR) Studies

 The FTIR spectrum of poly-o-anisidine (S-5), Sn(IV) arsenophosphate (S-1), and poly-o-anisidine Sn(IV) arsenophosphate (S-9), dried at 40°C, were taken by KBr disc method at room temperature.

### 2.8. Thermal (TGA and DTA) Studies

Simultaneous TGA and DTA studies of the composite cation-exchange material poly-o-anisidine Sn**(**IV) arsenophosphate (S-9) in original form were carried out by an automatic thermo balance on heating the material from 10°C to 1000°C at a constant rate (10°C per minute) in the air atmosphere (air flow rate of 200 mL min ^−1^).

### 2.9. Transmission Electron Microscopy (TEM) Studies

 Microphotographs of the composite material poly-o-anisidine Sn(IV) arsenophosphate (S-9) were obtained by the transmission electron microscope at various magnifications.

### 2.10. Selectivity (Sorption) Studies

The distribution coefficients (*K*_*d*_ values) of various metal ions on poly-o-anisidine Sn(IV) arsenophosphate composite were determined by batch method in various solvent systems. Various 200 mg portions of the composite cation-exchanger beads (S-9) in the H^+^ form were taken in Erlenmeyer flasks with 20 mL of different metal nitrate solutions in the required medium and kept for 24 hours with continuous shaking hours in a temperature controlled incubator shaker at 25 ± 2°C to attain equilibrium. The initial metal ion concentration was so adjusted that it did not exceed 3% of its total ion exchange capacity. The metal ions in the solution before and after equilibrium were determined by titrating against standard 0.005 molL^−1^ solution of EDTA as discussed by Reiliy et al. [8]. The alkali and alkaline earth metal ions [K^+^, Na^+^, Ca^2+^] were determined by flame photometry, and some heavy metal ions such as [Pb^2+^, Cd^2+^, Cu^2+^, Hg^2+^, Ni^2+^, Mn^2+^, Zn^2+^] were determined by atomic absorption spectrophotometry (AAS). The distribution quantity is the ratio of the amount of metal ions in the exchanger phase and in the solution phase. In other words, the distribution coefficient is the measure of a fractional uptake of metal ions competing for H^+^ ions from a solution by an ion-exchange material and hence mathematically can be calculated using the formula given as

(1) $$K_{d}=\frac{\text{m}\;\text{moles}\;\text{of}\;\text{metal}\;\text{ions}/\text{gm}\;\text{of}\;\text{ion-exchanger}}{\text{m}\;\text{moles}\;\text{of}\;\text{metal}\;\text{ions}/\text{mL}\;\text{of}\;\text{solution}}\left({\text{mLg}}^{-1}\right),$$

that is,

(2) $$K_{d}=\left[\frac{I-F}{F}\right]\times \frac{V}{M}\left({\text{mL}\;\text{g}}^{-1}\right),$$

where *I* is the initial amount of metal ion in the aqueous phase, *F* is the final amount of metal ion in the aqueous phase, *V* is the volume of the solution (mL), and *M* is the amount of cation-exchanger (g).

### 2.11. Preparation of Poly-o-anisidine Sn(IV) Arsenophosphate Cation Exchange Membrane

 The ion exchange membrane of poly-o-anisidine Sn(IV) arsenophosphate was prepared as discussed by Khan et al. [5] in earlier studies. To find out the optimum membrane composition, a different amount of the composite material was grounded to a fine powder and mixed thoroughly with a fixed amount (200 mg) of PVC in 10 mL tetrahydrofuran and 10 drops of dioctylphthalate. The resultant slurries were poured to cast in glass tubes of 10 cm in length and 5 mm in diameter. These glass tubes were left for slow evaporation for 24 hours. In this way four sheets of different thicknesses (0.2, 0.28, 0.3, and 0.4) mm were obtained. These sheets were cut in the shapes of discs using a sharp edge blade for further studies.

### 2.12. Characterization of Membrane

Physicochemical characterization is important to understand the performance of membrane. Thus some parameters such as porosity, water content, swelling, and thickness were determined as described elsewhere [9–13]. 

#### 2.12.1. Water Content (% Total Wet Weight)

First, the membranes were soaked into water to elute diffusible salt, blotted quickly with Whatmann filter paper to remove surface moisture and immediately weighed. These were further dried to a constant weight in a vacuum over P_2_O_5_ for 24 hours. The water content (total wet weight) was calculated as

(3) $$\%\;\text{Total}\;\text{wet}\;\text{weight}=\frac{W_{w}-W_{d}}{W_{w}}\times 100,$$

where *W*_*w*_ is the weight of the soaked/wet membrane, and *W*_*d*_ is the weight of the dry membrane.

#### 2.12.2. Porosity

The thickness of the membrane was measured by taking the average thickness of the membrane by using screw gauze. Swelling is measured as the difference between the average thicknesses of the membrane equilibrated with 1 molL^−1^ NaCl for 24 hours and the dry membrane:

(4) $$\text{Porosity}=\frac{W_{w}-W_{d}}{W_{w}\rho_{0}LA},$$

where *W*_*w*_ and *W*_*d*_ are weight of wet/soaked membrane, *ρ*_0_ is the density of water, while *A* and *L* are the area and thickness of the membrane.

#### 2.12.3. Thickness and Swelling

The thickness of the membrane was measured by taking the average thickness of the membrane by using screw gauze. Swelling is measured as the difference between the average thickness of the membrane equilibrated with 1 molL^−1^  NaCl for 24 hours and the dry membrane.

### 2.13. Fabrication of Ion-Selective Membrane Electrode

 The membrane sheet of 0.3 mm thickness as obtained by the above procedure was cut in the shape of disc and mounted at the lower end of a Pyrex glass tube (o.d. 0.8 cm, i.d. 0.6) with araldite. Finally the assembly was allowed to dry in air for 24 hours. The glass tube was filled with 0.1 molL^−1^ lead nitrate solution. A saturated calomel electrode was inserted in the tube for electrical contact, and another saturated calomel electrode was used as external reference electrode. The whole arrangement can be shown in Table 1.

Following parameters were evaluated to study the characteristics of the electrode such as lower detection limit, electrode response curve, response time, and working pH range.

### 2.14. Electrode Response or Membrane Potential

 To determine the electrode response, a series of standard solutions to be studied of varying concentrations were prepared. External electrode and ion selective membrane electrode are plugged in digital potentiometer, and the potentials were recorded.

For the determination of electrode potentials the membrane of the electrode was conditioned by soaking in 0.1 molL^−1^  Pb(NO_3_)_2_ solution for 5–7 days and for 1 hour before use. When electrode was not in use electrode must be kept in 0.1 molL^−1^ selective ion solution. Potential measurement was plotted against selected concentration of the respective ion in aqueous solution. 

### 2.15. Effect of pH

 A series of solutions of varying pH in the range of 1–11 were prepared, keeping the concentration of the relevant ion constant (1 × 10^−2^ molL^−1^). The value of electrode potential at each pH was recorded, and plot of electrode potential versus pH was plotted. 

### 2.16. The Response Time

The method of determining response time in the present work is being outlined as follows. The electrode is first dipped in a 1 × 10^−3^ molL^−1^ solution of the ion concerned and immediately shifted to another solution of 1 × 10^−2^ molL^−1^ ion concentration of the same ion (10 fold higher concentrations), and the solutions were continuously been stirred. The potential of the solution was read at zero second, just after dipping of the electrode in the second solution and subsequently recorded at the intervals of 5 seconds. The potentials were then plotted versus the time. The time during which the potentials attain constant value represents the response time of the electrode.

### 2.17. Selectivity Coefficient

To study the cationic interference due to other ions, the selectivity coefficients of various interfering cations for the ion-selective membrane electrode were determined by the mixed solution method as discussed elsewhere [9, 10]. A beaker of constant volume contains a mixed solution having a fixed concentration of interfering ion (M^n+^ ) (1 × 10^−2^ molL^−1^) and varying concentrations (1 × 10^−1^ to 1 × 10^−10^ molL^−1^) of the primary ions. Now the potential measurements were made using the membrane electrode assembly.

## 3. Results and Discussion

Poly-o-anisidine was synthesized by chemical oxidation using ammonium persulfate (APS) in 1 molL^−1^ HCl aqueous solution as discussed by Koval'chuk et al. [14]. The electron transfer phenomenon is considered as follows: 


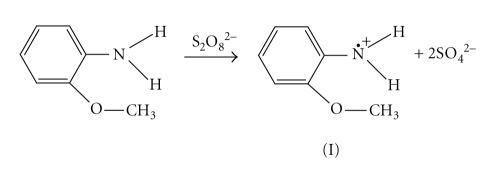


 Deprotonation of the primary cation radical can take place: 


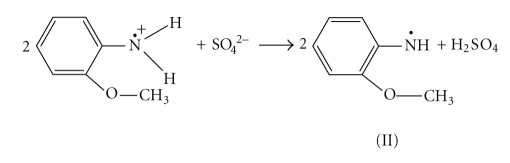


The “head-to-tail” formation of polymer can happen only when the isomerisation of the nitrenium radicals to quinoid structure (III) takes place:


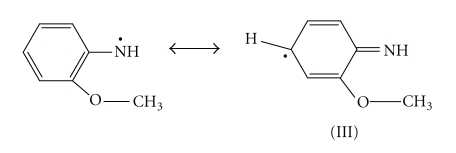


 The isomerisation of the o-anisidine occurs easily, while this process for the p-isomer is more complicated. The formation of the N–N bond takes place when dimer is formed by the head-to-head recombination of the two primary radicals. In this case the further chain propagation is impossible. The polymer formation proceeds through the interaction of primary formed and isomerized radicals and further through the oxidation of generated dimmers: 


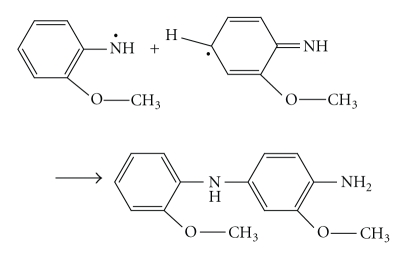


 In this case, it is assumed that transition of the quinoid structure into the link of benzoid type reisomerization takes place. For example, the trimer formation is possible as a result of the recombination of oxidized dimer and initial isomerised radical according to


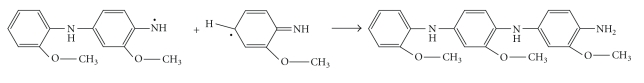


 This process can be defined as an oxidative polycondensation since the main-chain link and the molecule of initial monomer are not identical. Such propagation of polymer chains as a result of recombination of oligomeric species with the initial monomeric ones leads to the fast monomer consumption, as discussed elsewhere [15] during the oxidation of aniline in (NH_4_)_2_ · S_2_O_8_ in aqueous solution. The formation of inorganic precipitate of Sn(IV) arsenophosphate was significantly affected by the pH, and the most favorable pH of the mixture was ~1.0. The binding of poly-o-anisidine into the matrix of Sn(IV) arsenophosphate (assumed as X in the reaction) can be given as


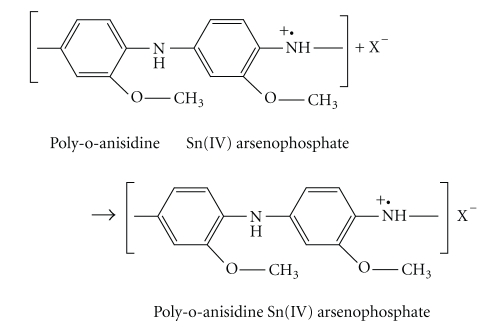


Various samples of organic-inorganic composite cation exchange material have been developed by the incorporation of electrically conducting polymer poly-o-anisidine into the inorganic matrices of granular Sn(IV) arsenophosphate. Due to high percentage of yield, better ion exchange capacity, reproducible behavior, and chemical and thermal stability, sample (S-9) in Table 2 was chosen for detailed studies. The percent composition of C, H, N, O, Sn, P, and As in the material was found to be 10.44, 1.8, 1.74, 35.79, 7.38, 5.59, and 37.25, respectively. 

**Figure 1:**
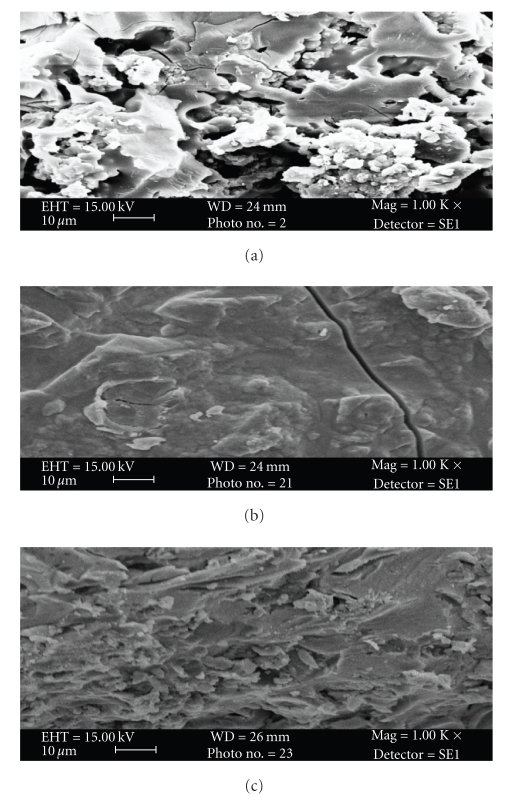
SEM photographs of poly-o-anisidine (a), Sn(IV) arsenophosphate (b) and poly-o-anisidine Sn(IV) arsenophosphate (c) cation exchangers.

**Figure 2:**
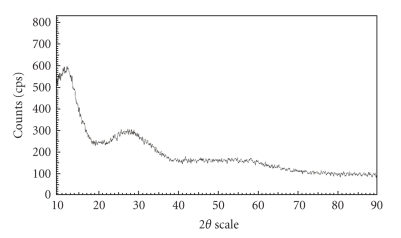
Powder X-ray diffraction pattern of poly-o-nisidine Sn(IV) arsenophosphate composite cation exchanger.

The scanning electron microphotographs of poly-o-anisidine (a), Sn(IV) arsenophosphate (b), and poly-o-anisidine Sn(IV) arsenophosphate (c) are given in Figure 1. It is clear from the photographs that after binding of organic polymer with inorganic precipitate of Sn(IV), arsenophosphate morphology has been changed, which indicates the formation of organic-inorganic composite cation exchanger poly-o-anisidine Sn(IV) arsenophosphate. The X-ray diffraction pattern of poly-o-anisidine Sn(IV) arsenophosphate cation exchange material (S-9) recorded in powdered sample exhibited some small peaks in the spectrum in Figure 2. It is clear from the figure that the nature of composite cation exchange material is semicrystalline. 

**Figure 3:**
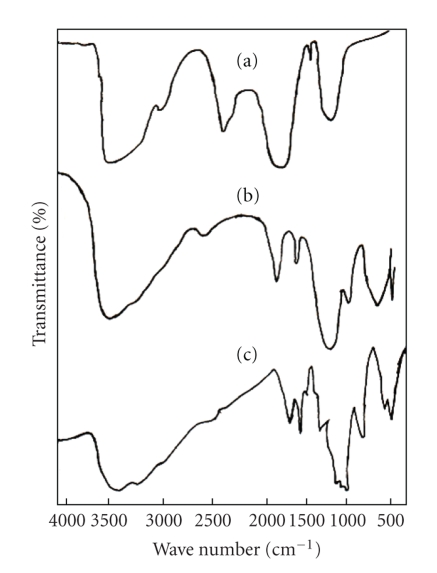
FTIR spectra of poly-o-anisidine (a) Sn(IV) arsenophosphate (b) and poly-o-anisidine Sn(IV) arsenophosphate composite cation exchanger (c).

 The IR peaks observed at 3421, 540, 587, 843, 1020, 1115, 1296, and 1590 cm^−1^ are the characteristic bands for Poly-o-anisidine Sn(IV) arsenophosphate composite cation exchanger (S-9) in Figure 3. A broad band at ~3421  cm^−1^ is due to the N–H stretching mode. The band at 1020 cm^−1^ is due to the orthosubstituted aromatic ring. Band stretching at 1590 and 1296 cm^−1^ shows the presence of C–N and C=N linkage as discussed by Ram et al. [6]. The band at   ~1115 cm^−1^ is attributed to a plane bending vibration of C–H which is formed during protonation of benzene ring. The two peaks at 587 and 540  cm^−1^ and a peak at 843 indicate the M–O bonding and Sn–O bonding as discussed by D. K. Singh and S. Singh [16]. 

**Figure 4:**
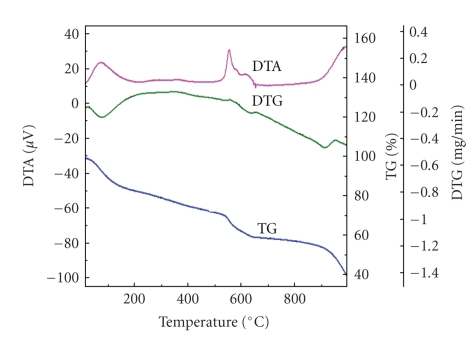
Simultaneous TGA-DTA curves of poly-o-anisidine Sn(IV) arsernophosphate composite cation exchanger.

 The thermogravimetric analysis curve of poly-o-anisidine Sn(IV) arsenophosphate composite material (S-9) shows fast weight loss (9.05%) up to 100°C due to the removal of external water molecules as discussed elsewhere [17, 18]. Slow weight loss of the material from 150°C to about 400°C may be due to the formation of pyrophosphate groups by the condensation of phosphate. Further, inclination point was observed at about 550°C which indicates the complete decomposition of the material and the formation of metal oxides. From about 600°C to 900°C, a sharp weight loss indicated by the curve may be due to the decomposition of the metal oxides. The DTA curves at 100, 550, and 1000°C show the exothermic decomposition reaction during the weight loss (Figure 4).

**Figure 5:**
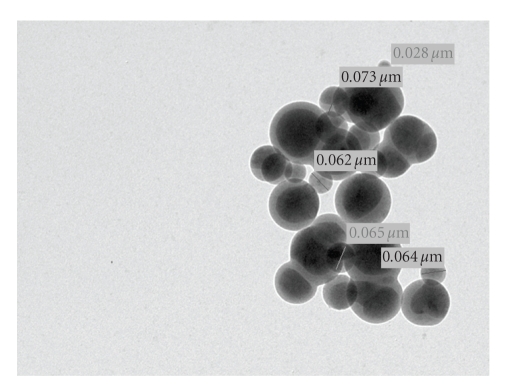
TEM Micrographs of Poly-o-anisidine Sn(IV) arsenophosphate composite cation exchanger at various magnifications.

 The transmission electron microphotographs of the composite material poly-o-anisidine Sn(IV) arsenophosphate (Figure 5) indicates that the particle size of the material is under nanorange .Thus the material is a nanocomposite material.

 In order to explore the potentiality of the present composite material (S-9) in the separation of metal ions, distribution studies for 15 metal ions were performed in 15 solvent systems observed from the data given in Table 3 that the *K*_*d*_-values vary with the composition and nature of the contacting solvents. Also it was found that Pb^2+^ was strongly adsorbed while Cu^2+^, Ca^2+^, Ba^2+^, and Th^2+^ were partially adsorbed on the surface of the ion exchanger. Thus, the studies showed that the material was found to be highly selective for Pb(II), which is an important environmental pollutant. In this work, nanocomposite poly-o-anisidine Sn(IV) arsenophosphate was also used for the preparation of heterogeneous ion-selective membrane electrode. 

 Sensitivity and selectivity of the ion-selective electrodes depend upon the nature of electroactive material, membrane composition, and physicochemical properties of the membranes employed. A number of samples of the poly-o-anisidine Sn(IV) arsenophosphate composite membranes were prepared with different amounts of composite and fixed amount (200 mg) of *PVC* and checked for the mechanical stability, surface uniformity, materials distribution, cracks and thickness, and so forth. 

 The results of thickness, swelling, porosity, and water content capacity of poly-o-anisidine Sn(IV) arsenophosphate composite cation exchange membrane are summarized in Table 4. The membrane sample M-3 (thickness 0.3 mm) was selected for further studies. Thus low order of water content, swelling, and porosity with less thickness of this membrane suggests that interstices are negligible, and diffusion across the membrane would occur mainly through the exchanger sites. 

**Figure 6:**
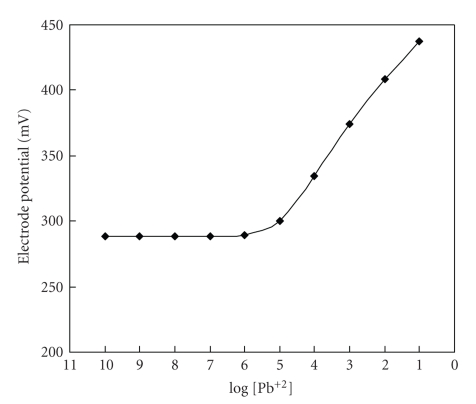
Calibration curve of poly-o-anisidine Sn(IV) arsenophosphate membrane electrode in aqueous solutions of Pb(NO_3_)_2_.

 The heterogeneous precipitate pb(II) ion selective membrane electrode obtained from poly-o-anisidine Sn(IV) arsenophosphate nanocomposite material gives linear response in the range 1 × 10^−1^ molL^−1^ and 1 × 10^−6^ molL^−1^ Figure 6. Suitable concentrations were chosen for sloping portion of the linear curve, and the slope value is found to be 32.8 mV. The limit of detection is determined from the intersection of the two extrapolated segments of the calibration graph as discussed elsewhere [19], it was found to be 1 × 10^−6^ molL^−1^, and thus the working concentration range is found to be 1 × 10^−1^  molL^−1^  to 1 × 10^−6^ molL^−1^ for Pb^2+^ ions. Below 1 × 10^−6^ molL^−1^ non linear response was observed that could be used for analytical applications as discussed elsewhere [20].

**Figure 7:**
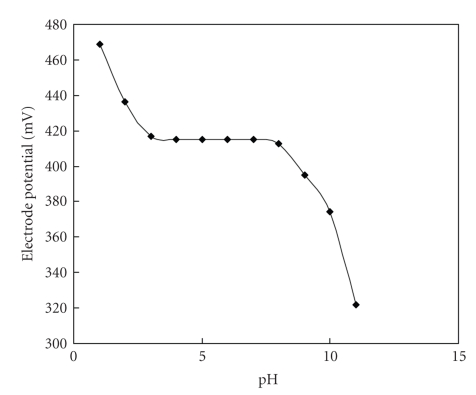
Effect of pH on the potential response of the poly-o-anisidine Sn(IV) arsenophosphate membrane electrode at 1 × 10^−2^ molL^−1^ Pb^2+^ concentration.

**Figure 8:**
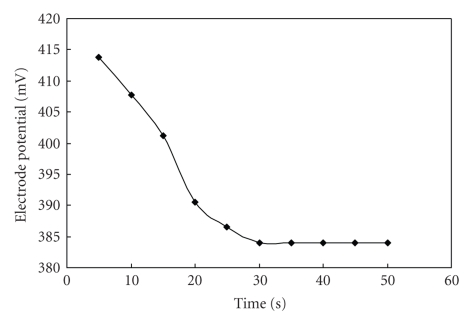
Time response curve of poly-o-anisidine Sn(IV) arsenophosphate membrane electrode.

 pH effects on the potential response of the electrode were measured for a fixed (1 × 10^−2^ molL^−1^) concentration of Pb^2+^ ions in different pH values. It is clear that electrode potential remains unchanged within the pH range 4.0–8.0 (Figure 7) known as working pH range for the electrode, below pH-4 the electrode is unable to work due to proton selectivity of the material, and above pH-8 there is possibility of formation of Pb(II) hydroxides. Another important factor is the promptness of the response of the ion-selective electrode. The average response time is defined as the time required for the electrode to reach a stable potential after successive immersion of the electrode in different Pb^2+^ ion solutions, each having a 10-fold difference in concentration. The response time in contact with 1 × 10^−2^ molL^−1^  Pb^2+^ ion solution was determined, and the results are shown in Figure 8. It is clear from the figure, that the response time of the membrane is ~30 seconds. 

The selectivity coefficients, *K*_Pb^2+^·M_^Por^ of various differing cations for the Pb(II) ion selective Poly-o-anisidine Sn(IV) arsenophosphate composite membrane electrode were determined, by the mixed solution method. The selectivity coefficient indicates the extent to which a foreign ion interferes with the response of the electrode towards its primary ions (Pb^2+^). The selectivity coefficient of various cations for the Pb(II) ion-selective poly-o-anisidine Sn(IV) arsenophosphate membrane electrode are given in Table 5 which suggest that the membrane electrode is selective for Pb(II) in presence of interfering ions.

Nano- and picomolar range pb(II) sensitive ion-selective electrodes are reported as discussed by Sokalski et al., Ceresa et al., Ngeontae et al., Sutter et al., and Pergel et al. [21–25] in literature. However poly-o-anisidine Sn(IV) arsenophosphate is a micromolar range Pb(II) sensitive membrane electrode, but its quick response time (~30 Seconds), working pH range (4–8), and thermal stability suggest some important aspects of such class of materials.

## 4. Conclusion

 Poly-o-anisidine Sn(IV) arsenophosphate is a nanocomposite ion exchange material exhibits that a good ion exchange capacity, thermal stability, and chemical resistivity. As shown in TEM photographs the particle sizes of the composite material are within the range of 0.073, 0.065, and 0.028 *μ*m. Thus the material can be considered as nanocomposite material. This material was also utilized as an electroactive component for the preparation of ion-selective membrane electrode for the determination of Pb(II) ions in aqueous solution. The membrane electrode showed a working concentration range 1 × 10^−1^ molL^−1^ to 1 × 10^−6^ molL^−1^, response time ~30 seconds, 4–8 pH range, and selectivity for Pb(II) in presence of other metal ions.

## Figures and Tables

**Table 1:** 

Internal reference electrode (SCE)	Internal electrolyte 0.1 M Pb^2+^	Membrane	Sample solution	External reference electrode (SCE)

**Table 2:** Conditions of preparation and the ion exchange capacity of poly-o-anisidine Sn(IV) arsenophosphate composite cation exchanger.

Sample	Mixing volume ratios (v/v)			pH of the inorganic ion exchanger	Mixing volume ratios (v/v)		Appearance of the samples	Ion exchange capacity in meq/g
	0.1 molL^−1^Na_2_HAsO_4_in DMW	0.1 molL^−1^ SnCl_4_ · 5H_2_O in 1 molL^−1^ HCl	0.1 molL^−1^ H_3_PO_4_ in DMW		2.5%CH_3_OC_6_H_4_NH_2_ in 1 molL^−1^ HCl	0.05 M (NH_4_)_2_S_2_O_8_ in 1 molL^−1^ HCl		
S-1	1	1	1	1	—	—	white granular	0.9
S-2	1	1	1	1	1	1	Blackish granular	0.99
S-3	1	1	2	1	1	1	Blackish granular	0.72
S-4	1.5	0.5	2	1	1	1	Blackish granular	0.45
S-5	—	—	—		1	1	Black powder	0.19
S-6	1	1	0.5	1	1	1	Blackish granular	0.5
S-7	1	1	0.5	1	1	1	Blackish granular	1.24
S-8	0.5	0.5	1	1	1	1	Blackish granular	1.2
S-9	1	1	0.5 (2 molL^−1^)	1	1	1	Blackish granular	1.82
S-10	0.5	0.5	1 (2 molL^−1^)	1	1	1	Blackish granular	0.52

**Table 3:** K_d_-values of some metal ions on poly-o-anisidine Sn(IV) arsenophosphate composite cation exchanger in different solvent systems.

Solvents	Metal ions														
	Sr^2+^	Ba^2+^	Pb^2+^	Ca^2+^	Hg^2^+	Mg^2+^	Th^4+^	Cu^2+^	Cd^4+^	Ni^2+^	Cr^3+^	Al^3+^	Fe^2+^	Ce^3+^	Co^4+^
DMW	172	26	400	32	60	88	165	139	271	100	200	38	50	250	66
0.1 molL^−1^ HCl	29	63	200	48	44	23	100	—	27	67	200	83	114	—	20
0.01 molL^−1^ HCl	54	122	766	112	44	38	38	543	59	15	67	29	29	322	85
0.001 molL^−1^ HCl	53	300	600	105	291	103	86	356	125	300	200	—	300	22	84
0.1 molL^−1^ HNO_3_	23	88	—	187	35	29	103	30	57	114	250	100	—	—	—
0.01 molL^−1^ HNO3	36	50	571	258	222	12	230	217	57	118	33	60	100	292	42
0.001 molL^−1^ HNO_3_	26	27	187	136	333	3	55	67	18	86	39	250	114	483	59
10% Formic acid	63	—	767	80	222	37	55	167	20	22	93	60	450	—	29
20% Acetone	178	575	733	189	11	30	74	367	137	267	29	19	800	657	8
0.1 molL^−1^ H_2_SO_4_	—	25	33	91	169	55	—	11	—	—	86	29	—	—	—
0.01 molL^−1^ H_2_SO_4_	11	171	556	136	500	26	96	74	181	33	67	48	86	517	186
10% Ethanol	126	250	1250	256	—	75	50	340	650	38	33	10	200	500	36
Buffer 10	59	133	70	60	93	850	—	84	—	—	67	59	145	—	—
0.1 molL^−1^ KCl	57	—	800	145	—	49	14	164	69	—	100	48	200	128	28
Ph 5.75	152	200	140	371	—	173	152	215	1033	—	—	280	86	—	34
															

**Table 4:** Characterization of ion exchange membranes of poly-o-anisidine Sn(IV) arsenophosphate.

Poly-o-anisidine Sn(IV) arseno phosphate composite material	Thickness of the membrane (mm)	Water content as % weight of wet membrane	Porosity	Swelling of % weightof wet membrane
M-1	0.2	2.0000	0.0005	0.04
M-2	0.28	2.1762	0.0003	0.03
M-3	0.3	2.15124	0.0003	0.03
*M-4*	0.4	2.96084	0.004	0.05

**Table 5:** Selectivity coefficient of various interfering metal ions of poly-o-anisidine Sn(IV) arsenophosphate composite cation exchanger membrane.

Interfering metal ions	Selectivity coefficient
Zn^+2^	2.4 × 10^−2^
Cd^+2^	2.2 × 10^−2^
Cu^+2^	2.3 × 10^−2^
Mg^+2^	2.0 × 10^−2^
Co^+2^	1.8 × 10^−1^
Na^+^	1.6 × 10^−1^
*K^+^*	1.4 × 10^−1^

## References

[1] Laine RM, Sanchez C, Brinker CJ, Giannelis E (1998). *Organic/Inorganic Hybrid Materials, Proceedings of the Symposium on Materials Research Society*.

[2] Klein LC, Francis LF, De Guire MR, Mark JE (1999). *Organic/Inorganic Hybrid Materials II, Proceedings of the Symposium on Materials Research Society*.

[3] Judeinstein P, Sánchez C (1996). Hybrid organic-inorganic materials: a land of multidisciplinarity. *Journal of Materials Chemistry*.

[4] Ruiz-Hitzky E, Casal B, Aranda P, Galván JC (2001). Inorganic-organic nanocomposite materials based on macrocyclic compounds. *Reviews in Inorganic Chemistry*.

[5] Khan AA, Inamuddin, Alam MM (2005). Determination and separation of Pb^2+^ from aqueous solutions using a fibrous type organic-inorganic hybrid cation-exchange material: polypyrrole thorium(IV) phosphate. *Reactive and Functional Polymers*.

[6] Ram MK, Carrara S, Paddeu S, Nicolini C (1997). Effect of annealing on physical properties of conducting poly(ortho-anisidine) Langmuir-Blodgett films. *Thin Solid Films*.

[7] Niwas R, Khan AA, Varshney KG (1999). Synthesis and ion exchange behaviour of polyaniline Sn(IV) arsenophosphate: a polymeric inorganic ion exchanger. *Colloids and Surfaces A*.

[8] Reiliy CN, Schmidt RW, Sadek FS (1959). *Journal of Chemical Education*.

[9] Amarchand S, Menon SK, Agrawal YK (1998). Water hardness determination using Mg(II) ion selective electrode. *Indian Journal of Chemical Technology*.

[10] Reilley CN, Schmid RW, Sadek FS (1959). Chelon approach to analysis (I) survey of theory and application. *Journal of Chemical Education*.

[11] Craggs A, Moody GJ, Thomas JDR (1974). PVC matrix membrane ion-selective electrodes: construction and laboratory experiments. *Journal of Chemical Education*.

[12] Srivastava SK, Jain AK, Agrawal S, Singh RP (1978). Studies with inorganic ion-exchange membranes. *Talanta*.

[13] Gregor HP, Jagobson H, Shair RC, Wetstone DM (1957). Interpolymer ion-selective membranes. I. Preparation and characterization of polystyrenesulfonic acid-Dynel membranes. *Journal of Physical Chemistry*.

[14] Koval'chuk EP, Stratan NV, Reshetnyak OV, Błazejowski J, Whittingham MS (2001). Synthesis and properties of the polyanisidines. *Solid State Ionics*.

[15] Mac Diarmid AG, Kaner RB, Skotheim TA (1986). *Handbook of Conducting Polymers*.

[16] Singh DK, Singh S (2004). Synthesis, characterization and analytical applications of zirconium(IV) sulphosalicylophosphate. *Indian Journal of Chemical Technology*.

[17] Khan AA, Alam MM (2003). Synthesis, characterization and analytical applications of a new and novel ‘organic-inorganic’ composite material as a cation exchanger and Cd(II) ion-selective membrane electrode: polyaniline Sn(IV) tungstoarsenate. *Reactive and Functional Polymers*.

[18] Khan AA, Inamuddin, Alam MM (2005). Preparation, characterization and analytical applications of a new and novel electrically conducting fibrous type polymeric-inorganic composite material: polypyrrole Th(IV) phosphate used as a cation-exchanger and Pb(II) ion-selective membrane electrode. *Materials Research Bulletin*.

[19] Amini MK, Mazloum M, Ensaf AA (1999). *Fresenius' Journal of Analytical Chemistry*.

[20] Demirel A, Dogan A, Canel E, Memon S, Yilmaz M, Kiliç E (2004). Hydrogen ion-selective poly(vinyl chloride) membrane electrode based on a
p-tert-butylcalix[4]arene-oxacrown-4. *Talanta*.

[21] Sokalski T, Ceresa A, Zwickl T, Pretsch E (1997). Large improvement of the lower detection limit of ion-selective polymer membrane electrodes. *Journal of the American Chemical Society*.

[22] Ceresa A, Bakker E, Hattendorf B, Gunther D, Pretsch E (2001). Potentiometric polymeric membrane electrodes for measurement of environmental samples at trace levels: new requirements for selectivities and measuring protocols, and comparison with ICPMS. *Analytical Chemistry*.

[23] Pergel E, Gyurcsanyi RE, Toth K, Lindner E (2001). Picomolar detection limits with current-polarized Pb^2+^ ion-selective membranes. *Analytical Chemistry*.

[24] Sutter J, Lindner E, Gyurcsanyi RE, Pretsch E (2004). A polypyrrole-based solid-contact Pb^2+^-selective PVC-membrane electrode with a nanomolar detection limit. *Analytical and Bioanalytical Chemistry*.

[25] Ngeontae W, Xu Y, Xu C (2007). Sensitivity and working range of backside calibration potentiometry. *Analytical Chemistry*.

